# Enhanced Gas Separation Performance of Carbon Molecular Sieve Membranes Derived from Polyimide/Polyaniline Blend Precursors

**DOI:** 10.3390/polym18172159

**Published:** 2026-09-04

**Authors:** Shiyang Zhang, Jiongcai Chen, Mingwei Cai, Linjie Qiu, Zengyao Zhang, Siyuan Chen, Rongtao Liu, Yonggang Min

**Affiliations:** 1 Institute of Chemistry, Henan Academy of Sciences, Zhengzhou 450046, China; syz131139@163.com (S.Z.); 15993738910@163.com (L.Q.); fendouhaofdh@163.com (S.C.); 2 School of Materials and Energy, Guangdong University of Technology, Guangzhou 510006, China; chenjiongcai@163.com (J.C.); caimw5@mail2.sysu.edu.cn (M.C.); 3 Jieyang High-Tech Industrial Development Zone Productivity Promotion Center, Jieyang 515561, China; gdjy_zzy@163.com; 4 School of Pharmacy, Hubei University of Science and Technology, Xianning 437100, China

**Keywords:** polyimide, polyaniline, carbon molecular sieve membrane, gas separation

## Abstract

Polyimide membranes are generally limited by the permeability–selectivity trade-off during gas separation, which significantly restricts their further development and practical applications. Carbon molecular sieve membranes derived from polyimide (PI) precursors via pyrolysis have attracted considerable attention due to their excellent molecular sieving capability. In this study, polyaniline (PANI) was introduced to modify the polyimide matrix, and a series of PI/PANI precursor membranes with different PANI loadings were fabricated, followed by pyrolysis to obtain the corresponding CMS/PANI membranes. The effects of PANI incorporation on the structural evolution and gas separation performance of the membranes were systematically investigated. The results demonstrated that PANI incorporation effectively increased the fractional free volume (FFV) of the precursor membranes and promoted the formation of microporous structures during pyrolysis, resulting in enhanced BET surface area and a more developed microporous network in CMS/PANI membranes. Compared with the pristine PI-derived CMS membrane, CMS/PANI membranes exhibited significantly improved gas permeability while maintaining comparable gas pair selectivity. Among the prepared membranes, CMS/PANI-20 achieved the optimal overall separation performance, with H_2_ and CO_2_ permeabilities of 2012 and 754 Barrer, respectively, and H_2_/CH_4_ and CO_2_/CH_4_ selectivities of 253 and 95, respectively, exceeding the corresponding Robeson upper bounds.

## 1. Introduction

With the increasing global energy demand and growing concerns over carbon emissions, the development of highly efficient and energy-saving gas separation technologies has attracted significant attention, particularly for hydrogen purification and CO_2_ capture applications [1,2,3]. Compared with conventional separation processes, membrane technology offers advantages of low energy consumption, simple operation, and continuous processing, making it one of the most promising approaches for efficient gas separation [4]. However, conventional polymeric membranes remain constrained by the intrinsic permeability–selectivity trade-off, where improvements in permeability are often achieved at the expense of selectivity, limiting their practical applications in industrial gas separation. This permeability–selectivity trade-off is commonly described using an empirical upper-bound relationship, which provides a benchmark for evaluating the separation performance of polymeric membrane materials. The original Robeson upper bounds have subsequently been updated for several gas pairs as higher-performance membrane materials have emerged [5]. Carbon molecular sieve membranes (CMSMs), derived from the pyrolysis of polymer precursors, have emerged as promising candidates to overcome these limitations due to their rigid carbon frameworks, tunable microporous structures, and excellent plasticization resistance [6,7]. By rationally tailoring precursor structures and controlling carbon structure evolution during pyrolysis, the pore architecture and gas separation performance of CMS membranes can be effectively optimized [8].

Among various polymer precursors, polyimide (PI) has been widely employed for the fabrication of CMS membranes owing to its excellent thermal stability, mechanical strength, chemical resistance, and favorable film-forming capability [9]. However, PI-derived CMS membranes often undergo excessive carbon densification during pyrolysis, which restricts the development of effective microporous structures and gas transport pathways [10,11,12]. In recent years, the incorporation of fillers into polymer matrices to construct mixed matrix membranes has been regarded as an effective strategy for enhancing gas separation performance. Researchers have introduced various inorganic fillers into polymer precursors to regulate the microstructure of CMS membranes. Zhang et al. [13] incorporated ZSM-5 into polyimide precursors and fabricated CMS/ZSM-5 mixed matrix membranes through subsequent pyrolysis. The introduction of ZSM-5 increased the porosity of the carbon membrane and facilitated gas transport. As a result, the PIZ20-700 membrane exhibited H_2_ permeabilities approximately 7 and 6.5 times higher than that of the pristine CMS membrane under pure-gas and mixed-gas conditions, respectively, while achieving enhanced H_2_/CO_2_ separation performance. Cai et al. [14] employed KH-550-treated 10X zeolite as a functional filler in PI precursors and fabricated CMS/10X mixed matrix membranes through pyrolysis at 650 °C. CMS/10X-7.5% showed the best overall separation performance, with an H_2_ permeability of 1709 Barrer and H_2_/CH_4_ and H_2_/N_2_ selectivities of 551 and 105, respectively. Moreover, the membrane maintained excellent stability during cyclic testing. Jiao et al. [15] introduced ZIF-108 into PI P84 precursors to fabricate MOF-derived carbon molecular sieve membranes. The incorporation of ZIF-108 promoted the formation of ultramicroporous structures and regulated the pore size distribution, leading to enhanced H_2_ permeability and a 75% increase in H_2_/N_2_ selectivity, thereby effectively improving the separation performance of carbon membranes. Li et al. [16] prepared PI-based CMS membranes by blending cellulose acetate (CA) with PI and regulating the carbon structure through CA carbonization. The introduction of 10% CA optimized the carbon framework and enhanced gas transport properties. The resulting CMS membrane achieved an H_2_ permeability of 5300 Barrer with an H_2_/N_2_ selectivity of 42, demonstrating the effectiveness of polymer blending in tailoring CMS membrane performance.

Polyaniline (PANI) is a nitrogen-containing conjugated polymer with an aromatic structure, low cost, good chemical stability, and excellent carbonization capability, making it a promising functional polymer for carbon material fabrication [17,18,19]. Compared with inorganic fillers, PANI can form more homogeneous blends with polymer matrices and regulate the chain packing behavior of polymers, thereby influencing the free volume fraction of precursor membranes. Moreover, during pyrolysis, PANI can facilitate the formation of nitrogen-doped carbon structures and microporous architectures, providing additional transport pathways for gas molecules. However, the role of PANI as a polymeric blending component in simultaneously regulating the precursor free volume and the pore structure evolution of PI-derived CMS membranes has not been sufficiently investigated. In particular, the relationship between PANI-induced changes in precursor chain packing and the subsequent development of microporous structures during pyrolysis remains unclear.

In this study, PMDA-ODA polyimide, with a repeating-unit formula of (C_22_H_10_N_2_O_5_)_n_, was blended with PANI, with a nominal repeating-unit formula of (C_6_H_5_N)_n_, to prepare PI/PANI precursor membranes, which were subsequently converted into CMS/PANI membranes through pyrolysis at 650 °C. This work aimed to elucidate how PANI incorporation regulates the free volume of PI/PANI precursors and the pore structure evolution of the resulting CMS membranes and thereby affects their gas separation performance. The results demonstrated that the incorporation of an appropriate amount of PANI effectively increased the free volume of precursor membranes and promoted the formation of microporous and ultramicroporous structures during pyrolysis.

## 2. Materials and Methods

### 2.1. Materials

4,4′-Oxydianiline (ODA, C_12_H_12_N_2_O, 99%), pyromellitic dianhydride (PMDA, C_10_H_2_O_6_, 99%), polyaniline (PANI, 99%), and N,N-dimethylacetamide (DMAc, C_4_H_9_NO, 99%) were purchased from Shanghai Macklin Biochemical Co., Ltd (Shanghai, China). All chemicals were used as received without further purification.

### 2.2. Preparation of PI/PANI Membranes

A series of PI/PANI blend membranes with different PANI contents (0, 5, 10, 15, 20, and 25 wt%) were prepared. First, 0.675 g of 4,4′-oxydianiline (ODA) was dissolved in 8.075 g of N,N-dimethylacetamide (DMAc) under continuous stirring for more than 30 min until a homogeneous solution was obtained. Subsequently, 0.75 g of pyromellitic dianhydride (PMDA) was added to the above solution, and the mixture was stirred at 300 rpm for 12 h at 25 °C to form a poly(amic acid) (PAA) solution. Meanwhile, a predetermined amount of PANI powder was dispersed in 5.425 g of DMAc and stirred at 300 rpm for 30 min to achieve a uniform PANI dispersion. The PANI dispersion was slowly added to the PAA solution and further stirred for 10 min to obtain a homogeneous PAA/PANI mixture. The resulting mixture was cast onto a clean glass plate using a casting rod to prepare uniform and smooth membranes. The obtained PAA/PANI films were dried in an oven at 80 °C for 30 min. Subsequently, the PAA/PANI membranes were thermally imidized at 260 °C for 20 min and then further treated at 360 °C for 20 min. After cooling to room temperature, PI/PANI blend membranes were successfully obtained.

### 2.3. Preparation of CMS/PANI Membranes

The pre-fabricated PI/PANI membranes were cut into circular samples with a diameter of 10 mm and subsequently placed in a tubular furnace. The membranes were pyrolyzed under a continuous N_2_ flow of 80 mL min^−1^ by heating from 25 °C to 650 °C at a heating rate of 5 °C·min^−1^. After reaching 650 °C, the samples were maintained at this temperature for 120 min and then naturally cooled to room temperature under a N_2_ atmosphere to prevent oxidation of the carbon framework. The resulting carbon molecular sieve membranes were denoted as CMS/PANI-X, where X represents the mass fraction of PANI in the precursor membrane. For example, the CMS membrane derived from a PI/PANI precursor containing 5% PANI was designated as CMS/PANI-5. The CMS/PANI membranes exhibited an average thickness of approximately 51 μm, with an effective testing area of about 0.120 cm^2^.

### 2.4. Gas Permeation and Stability Measurements of Separation Membranes

The gas permeation properties of the prepared membranes toward H_2_, N_2_, CH_4_, and CO_2_ were evaluated using a constant-volume/variable-pressure time-lag method. The gas permeation measurement setup used in this study was self-constructed according to a previously reported procedure [20]. All gas permeation tests were performed at 35 °C under an upstream pressure of 2 bar. The permeability (P) of each gas was calculated according to Equation (1):

(1) $$P=D\times S=10^{10}\times \frac{V_{d}\times l}{p_{up}\times T\times R\times A}\times \frac{dp}{dt}$$

where P represents the gas permeability of the membrane (Barrer), D is the diffusion coefficient (cm^2^·s^−1^), and S is the solubility coefficient (cm^3^ (STP)·cm^−3^·cmHg^−1^). V_d_ represents the calibrated permeated gas volume (cm^3^), l is the average membrane thickness (cm), p_up_ is the upstream pressure (cmHg), T is the testing temperature (308 K), A is the effective membrane area (cm^2^), R is the gas constant (0.278 cm^3^·cmHg·cm^−3^(STP)·K^−1^), and dp/dt represents the pressure increase rate at the downstream side (cmHg·s^−1^).

### 2.5. Characterization

The crystal structures of the membrane samples were characterized by X-ray diffraction (XRD) using a D/MAX-Ultima IV (Tokyo, Japan) diffractometer equipped with Cu Kα radiation (λ = 0.15406 nm). The diffraction patterns were collected over a 2θ range of 5–60° at a scanning rate of 10°·min^−1^. The textural properties of the membranes were investigated by N_2_ adsorption–desorption measurements at 77 K using an ASAP-2460 automatic surface area analyzer (Micromeritics Instrument Corporation, Norcross, GA, USA). The specific surface areas were calculated based on the Brunauer–Emmett–Teller (BET) method, and the pore size distributions were determined using the non-local density functional theory method. Fourier transform infrared spectroscopy (FTIR) measurements were performed using a Nicolet ATR-60 spectrometer (Thermo Fisher Scientific, Madison, WI, USA) to analyze the chemical bonding structures and functional groups of the samples. The thermal decomposition behaviors of the membranes were evaluated by thermogravimetric analysis (TGA) using a TGA/DSC3+ instrument (TGA/DSC3+ instrument). The measurements were conducted from 35 to 800 °C at a heating rate of 10 °C·min^−1^ under a nitrogen atmosphere.

### 2.6. Molecular Simulation and FFV Calculation

Molecular simulations were performed using Materials Studio 2020. Polymer chains containing 10 repeating units were first constructed, and energy minimization was performed using the Forcite module with the COMPASS force field. The polymer chains were then randomly packed into a cubic amorphous cell with dimensions of 4.1 × 4.1 × 4.1 nm^3^ using the Amorphous Cell module. The constructed cells were subjected to geometry optimization, followed by NPT molecular dynamics simulation at 0.5 GPa for 40 ps and subsequent relaxation under the NPT ensemble for 500 ps. The simulation cells were then annealed from 300 to 700 K under the NPT ensemble for five cycles over a total simulation time of 800 ps, followed by an additional 500 ps relaxation. Finally, a 700 ps NVT simulation was performed to obtain the equilibrated structures. The fractional free volume (FFV) and free-volume distribution were analyzed using the atom volumes and surfaces tool.

The FFV was calculated according to the following equation:

FFV = (V_cell_ − V_occupied_)/V_cell_

where *V_cell_* is the total volume of the simulation cell, and *V_occupied_* is the occupied volume of the polymer chains.

## 3. Results and Discussion

**Figure 1:**
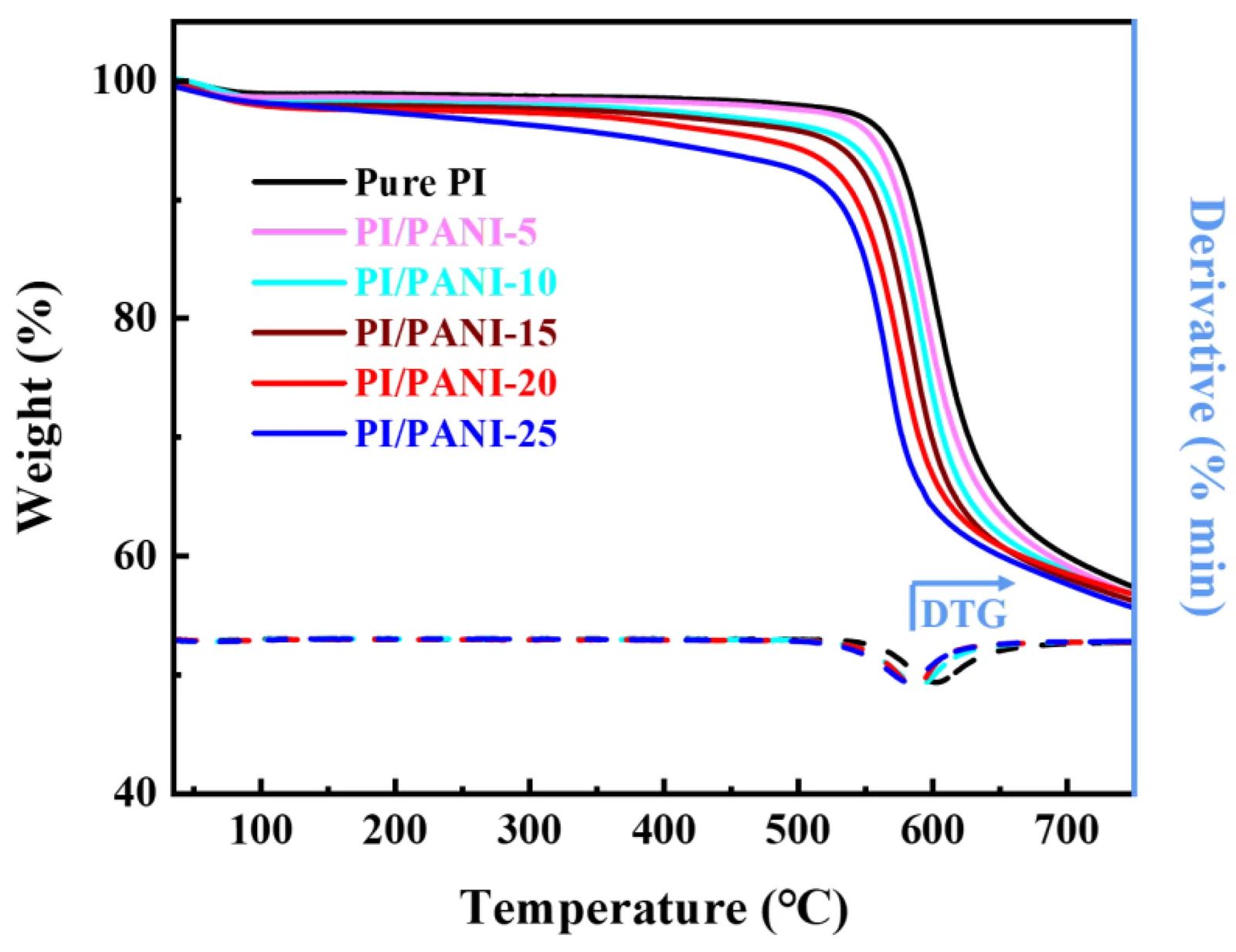
TGA and DTG curves of PI/PANI blend membranes with different PANI loadings.

Figure 1 displays the TGA and DTG curves of PI/PANI blend membranes containing different PANI contents. As shown in Figure 1, when the temperature increased from 510 to 650 °C, a sharp weight loss was observed, which was primarily associated with the thermal degradation and carbonization of the PANI backbone. During this stage, the weight loss reached approximately 36.2%. Upon further heating, the decomposition rate gradually decreased, suggesting the completion of the major decomposition reactions. The subsequent weight stabilization could be related to the occurrence of aromatic dehydrogenation, cross-linking, and condensation reactions, which promote the transformation of polymer chains into a stable carbonaceous framework [21]. Above 650 °C, the PI/PANI precursor membranes underwent extensive structural transformation, accompanied by the decomposition of unstable polymer segments and the formation of amorphous carbon structures. This observation confirms the successful conversion of PI/PANI precursors into carbon molecular sieve membranes (CMS/PANI). It is worth noting that the incorporation of PANI slightly reduced the thermal decomposition temperature of the PI/PANI blend membranes. This behavior can be attributed to the relatively lower thermal stability of PANI compared with PI, leading to accelerated decomposition at elevated temperatures. Furthermore, the decrease in thermal stability became more evident with increasing PANI loading. Despite this reduction, all six PI/PANI precursor membranes exhibited carbon yields higher than 56% at 650 °C.

**Figure 2:**
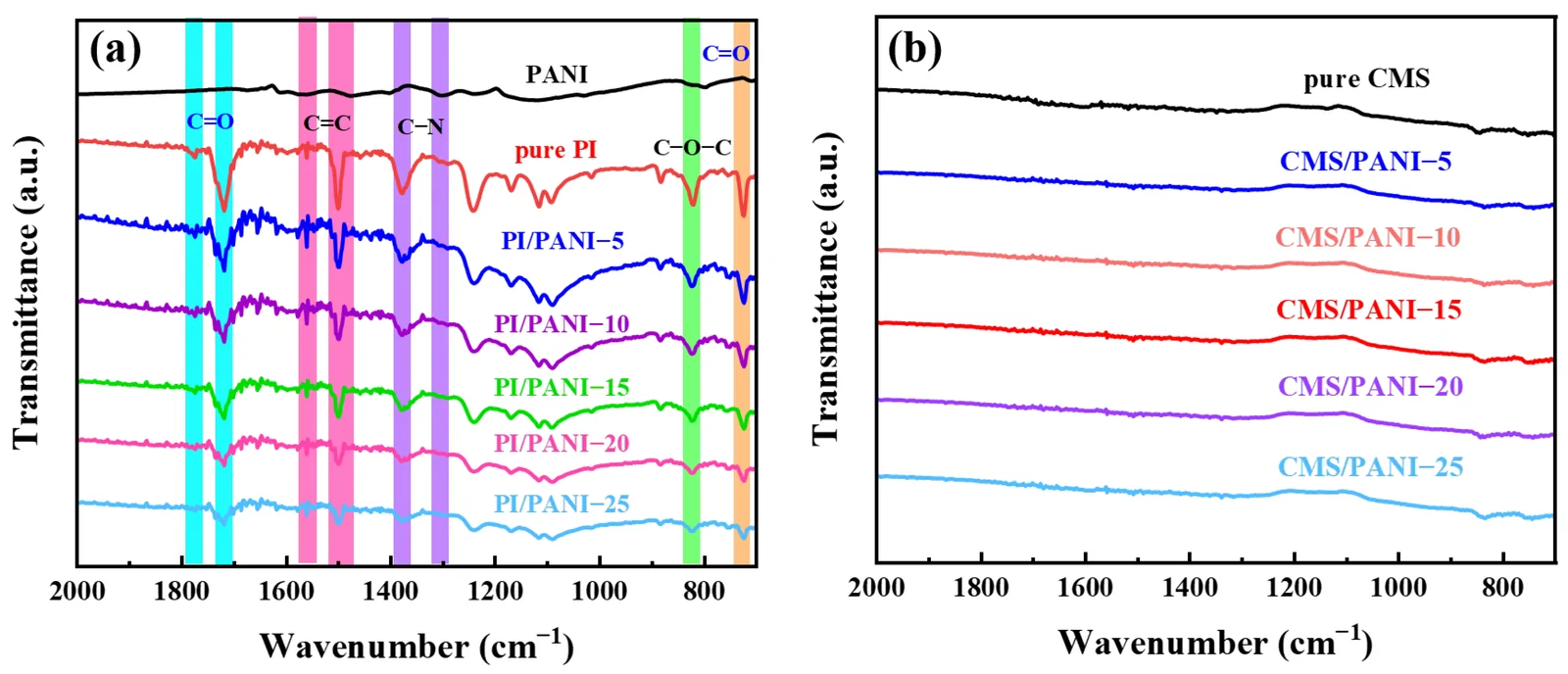
FTIR spectra of (**a**) precursors with different PANI loading and (**b**) carbon molecular sieve membranes.

The chemical structures of pristine PANI, PI/PANI blend membranes, and the derived carbon molecular sieve membranes were investigated by FTIR spectroscopy, as shown in Figure 2. As displayed in Figure 2a, the characteristic peaks located at 1560 and 1480 cm^−1^ for pristine PANI were assigned to the stretching vibrations of C=C bonds in the quinoid and benzenoid rings, respectively [22]. The peak at 1280 cm^−1^ was attributed to the C-N stretching vibration of the PANI backbone, confirming the preservation of the characteristic structure of PANI [23]. For all PI/PANI blend membranes, the characteristic absorption bands at 1776 and 1720 cm^−1^ corresponded to the asymmetric and symmetric stretching vibrations of the imide carbonyl groups (C=O), respectively [24]. The peaks at 1560 and 1500 cm^−1^ were associated with the stretching vibrations of aromatic C=C bonds in the PI backbone. The absorption band at 1375 cm^−1^ was attributed to the stretching vibration of C-N bonds [25], while the peaks at 1240, 820, and 720 cm^−1^ were assigned to the stretching vibration of C-O-C bonds and the bending vibration of imide carbonyl groups, respectively. The presence of these characteristic absorption bands confirms the successful formation of the PI structure. With increasing PANI loading, the intensities of the characteristic C=O peaks at 1776 and 1720 cm^−1^ gradually decreased, indicating the successful incorporation of PANI into the PI matrix and the formation of PI/PANI blend membranes. The FTIR spectra of all CMS/PANI membranes are presented in Figure 2b. After pyrolysis, all characteristic absorption peaks associated with PI and PANI disappeared, suggesting the decomposition of the original polymer structures and their transformation into carbonaceous frameworks during the carbonization process [26].

**Figure 3:**
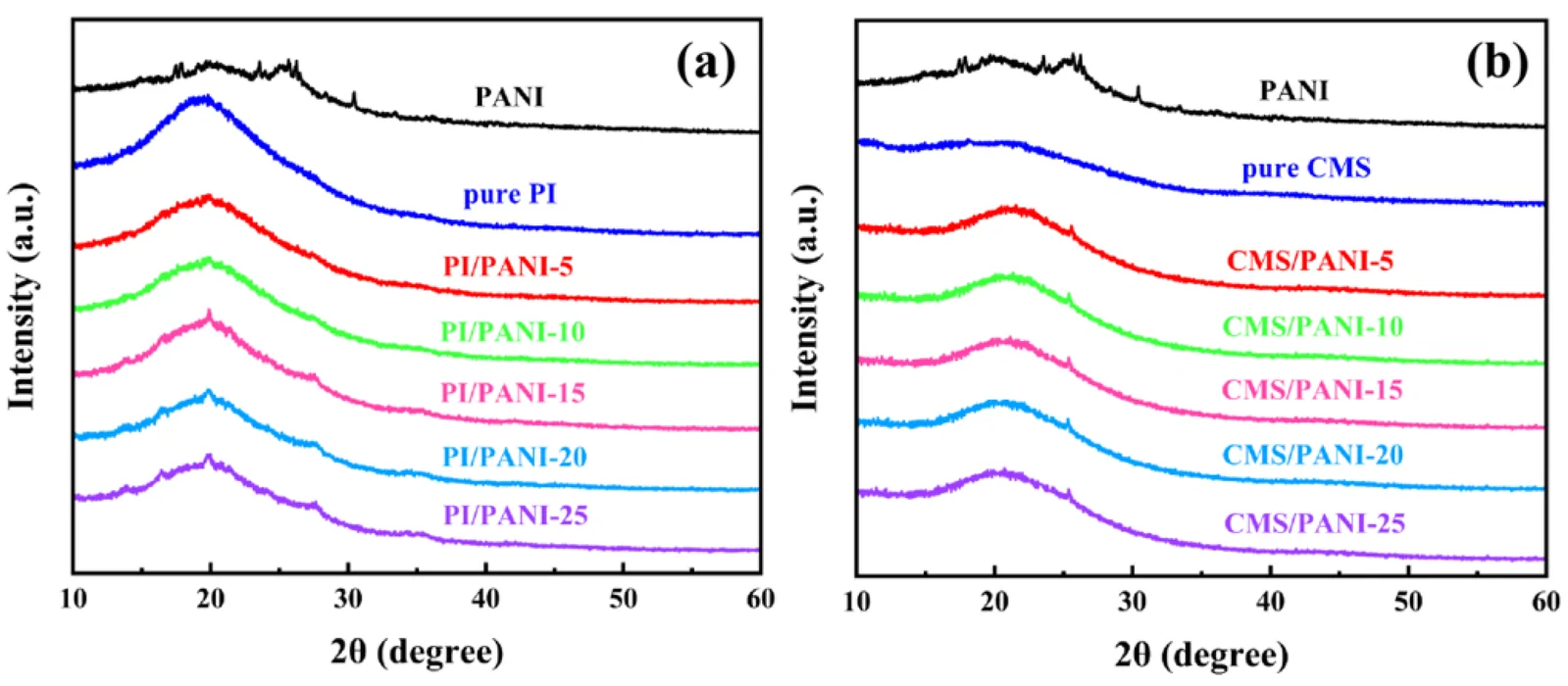
XRD curves of (**a**) PI precursors and (**b**) carbon molecular sieve membranes with different PANI content.

Figure 3 presents the X-ray diffraction (XRD) patterns of pristine PANI, PI/PANI blend membranes, and the corresponding derived CMS membranes. As shown in Figure 3a, all PI/PANI blend membranes exhibited a broad diffraction feature in the range of 20–30°, indicating the predominantly amorphous structure of the PI matrix [27]. With increasing PANI content, several weak diffraction features gradually became distinguishable from the broad amorphous background, particularly for PI/PANI-15, PI/PANI-20, and PI/PANI-25. These weak features are consistent with those observed for pristine PANI and with the characteristic diffraction behavior of PANI reported in the literature [28], indicating an increasing contribution of PANI-related structural features at higher PANI contents. As shown in Figure 3b, all CMS/PANI membranes exhibited a broad diffraction peak centered in the range of 20–30° without distinct sharp diffraction peaks, confirming their predominantly amorphous carbon structure. The overall XRD profiles of the CMS/PANI membranes remained similar after PANI incorporation, suggesting that the addition of PANI did not induce pronounced long-range crystalline ordering during pyrolysis.

**Figure 4:**
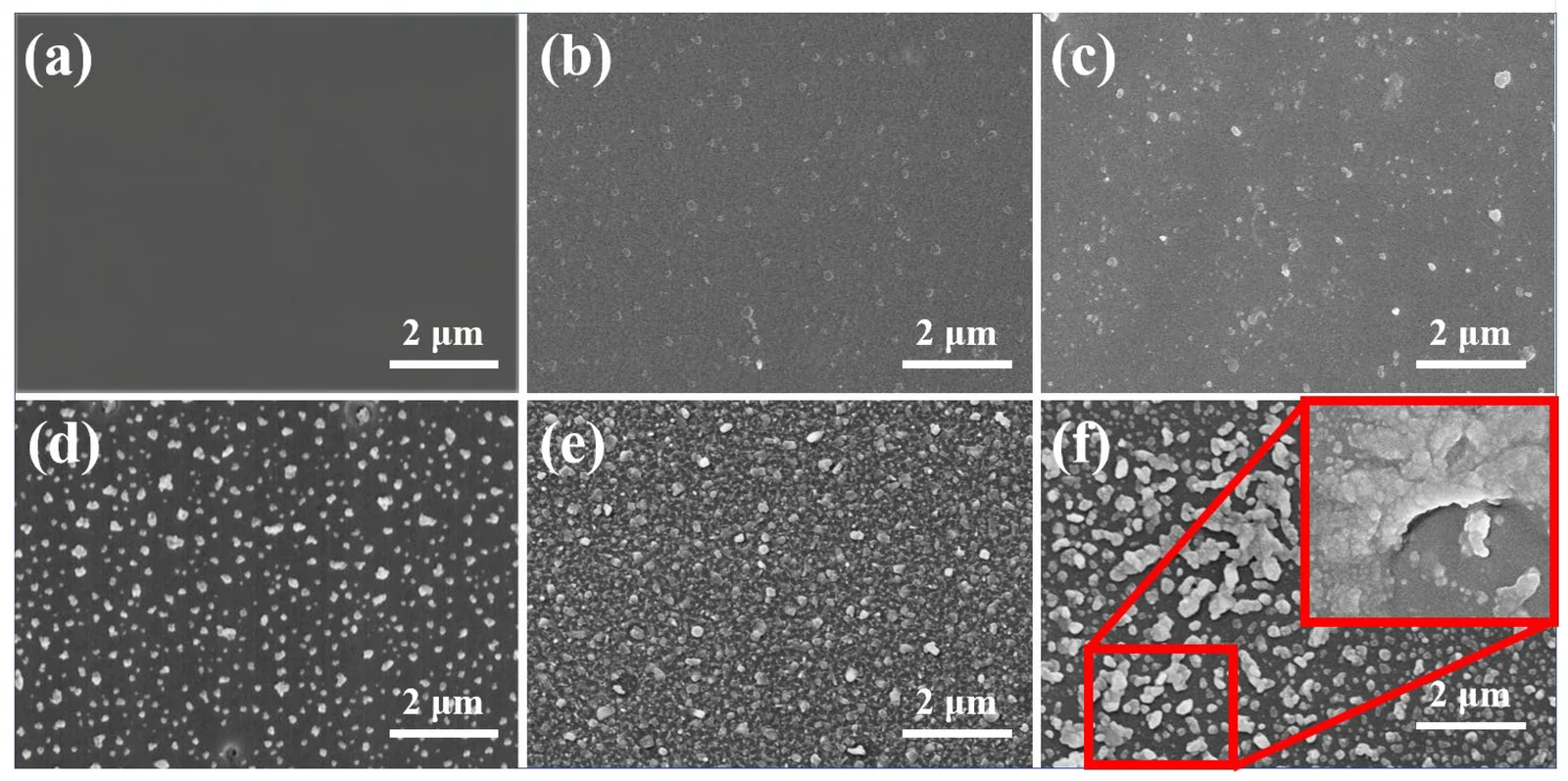
SEM images of (**a**) pure CMS, (**b**) CMS/PANI-5, (**c**) CMS/PANI-10, (**d**) CMS/PANI-15, (**e**) CMS/PANI-20, and (**f**) CMS/PANI-25.

Figure 4a–f show the surface morphologies of CMS membranes with different PANI contents. The pure CMS membrane exhibited a relatively smooth and compact surface without obvious defects or voids. With increasing PANI content, particle-like features gradually appeared and became more pronounced on the membrane surface. For CMS/PANI-5 to CMS/PANI-20, these features were relatively uniformly distributed over the carbon matrix, indicating a comparatively homogeneous membrane morphology. However, when the PANI content increased to 25 wt%, obvious aggregation of the particle-like features was observed, accompanied by the formation of larger surface defects and voids, as highlighted in the enlarged SEM image. Such morphological heterogeneity may generate non-selective transport pathways and thus adversely affect the gas separation selectivity of the membrane.

**Figure 5:**
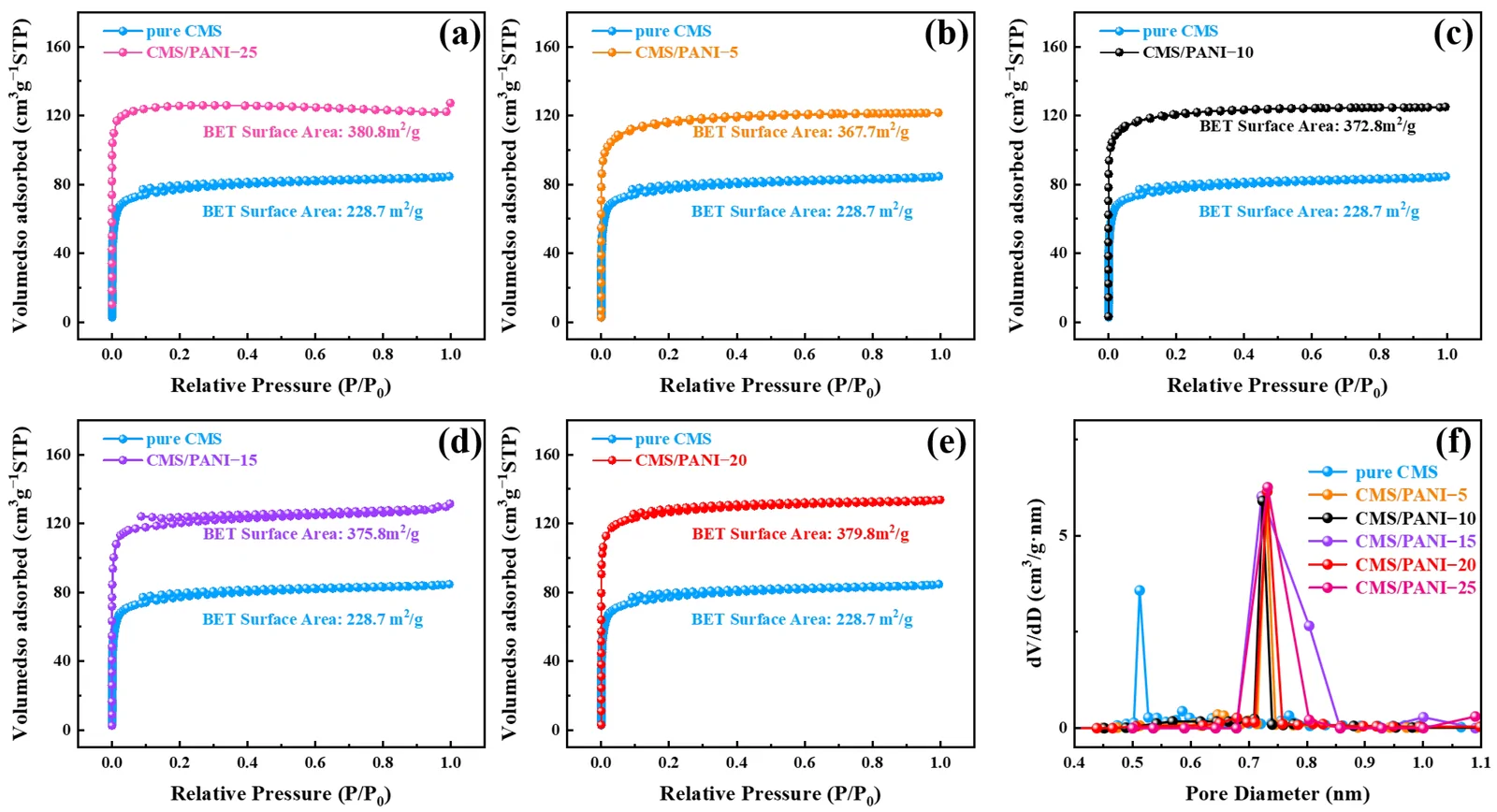
Adsorption–desorption isotherms of (**a**) CMS/PANI-0 and CMS/PANI-5, (**b**) CMS/PANI-0 and CMS/PANI-10, (**c**) CMS/PANI-0 and CMS/PANI-15, (**d**) CMS/PANI-0 and CMS/PANI-20 and (**e**) CMS/PANI-0 and CMS/PANI-25 as well as their pore size distributions (**f**).

To elucidate the effect of high-temperature pyrolysis on the pore structure evolution of the derived carbon molecular sieve membranes, N_2_ adsorption–desorption measurements were conducted to investigate the porous characteristics of CMS/PANI-X membranes, as shown in Figure 5. As observed from the adsorption–desorption isotherms, the introduction of PANI significantly modified the porous structure of the CMS membranes. Specifically, after incorporating 5% PANI into the PI precursor, the BET surface area of the resulting CMS membrane increased from 228.7 to 367.7 m^2^·g^−1^. The enhanced surface area can be attributed to the different thermal decomposition behaviors of PANI and PI. Due to its lower thermal stability, PANI undergoes decomposition at lower temperatures during pyrolysis, leading to the elimination of unstable polymer segments and the generation of additional free volume within the carbon matrix. Consequently, the decomposition of PANI provides additional pathways for pore development and promotes the formation of a more open porous structure. With further increasing PANI loading, the BET surface area of CMS/PANI membranes exhibited a gradual upward trend, increasing from 367.7 m^2^·g^−1^ for CMS/PANI-5 to 380.8 m^2^·g^−1^ for CMS/PANI-25 (Figure 5a–e). In comparison with the pristine PI-derived CMS membrane, all PI/PANI-derived CMS membranes exhibited increased micropore volumes after carbonization, accompanied by a shift in the pore size distribution toward larger microporous regions. Such structural evolution is expected to provide more accessible diffusion pathways, thereby facilitating gas transport through the CMS membranes [11,29]. Moreover, the micropore volume continuously increased with increasing PANI content. Generally, the development of microporous structures can provide additional adsorption and diffusion sites, contributing to enhanced gas permeability. Furthermore, CMS/PANI membranes exhibited a broader pore size distribution, which may originate from the increasing fraction of thermally unstable PANI domains. During pyrolysis, these domains can evolve from isolated regions into interconnected carbon channels, ultimately generating a co-continuous carbon framework [30].

**Figure 6:**
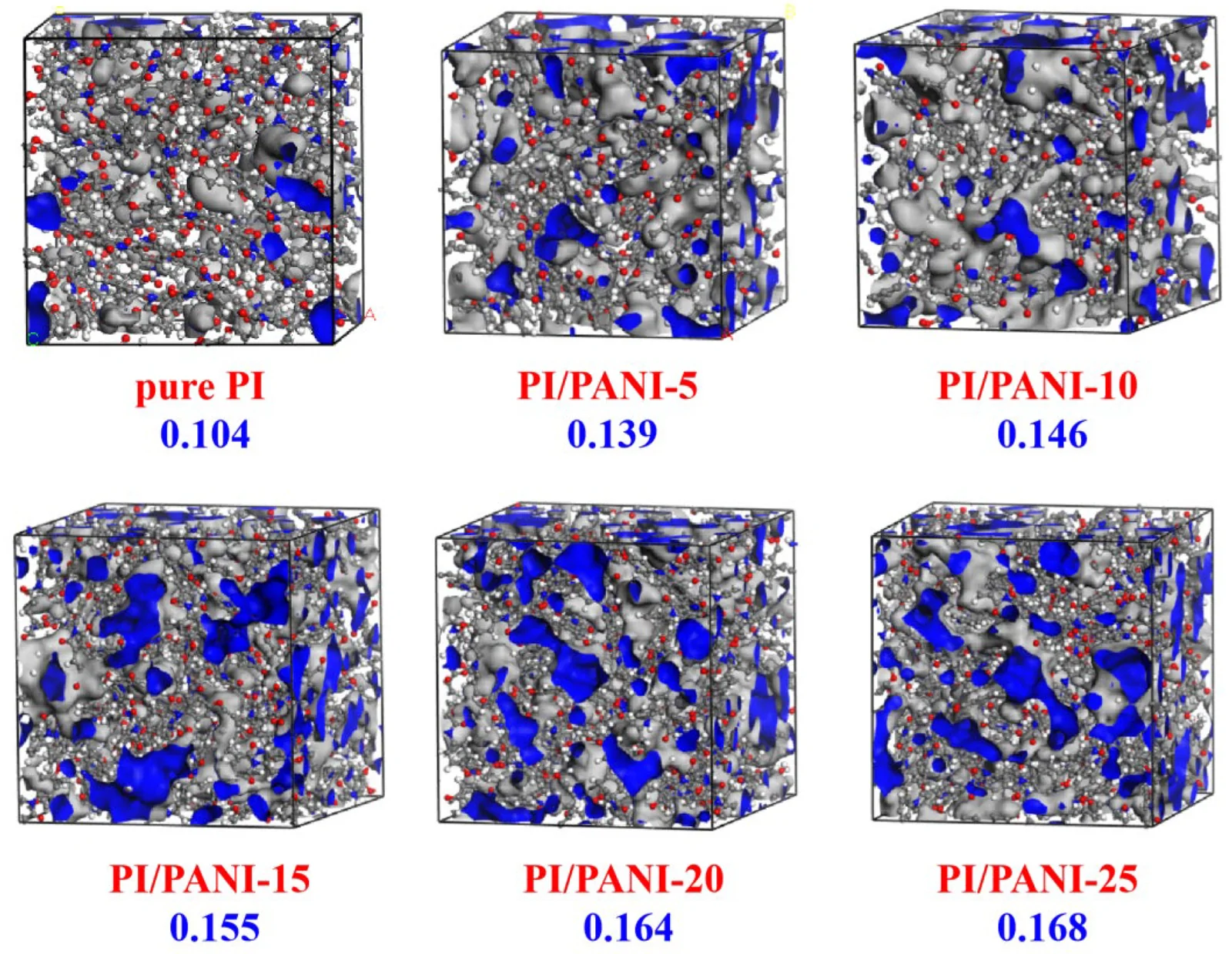
Simulated three-dimensional structures and fractional free volume (FFV) of PI/PANI membranes with different PANI loadings.

To further elucidate the relationship between the molecular structure of PI/PANI precursors and their gas separation properties, the fractional free volume (FFV) values of polymer membranes with different PANI contents were estimated through theoretical calculations, as shown in Figure 6. A clear variation in FFV was observed after incorporating PANI into the PI matrix. The pristine PI membrane exhibited the lowest FFV value of only 0.104. After introducing 5% PANI, the FFV of the PI/PANI blend membrane increased significantly to 0.139, corresponding to an enhancement of approximately 34%. With further increasing the PANI loading, the FFV values of the PI/PANI blend membranes continuously increased, indicating that the incorporation of PANI effectively disrupted the compact packing of PI polymer chains and generated additional intermolecular free volume [31]. This phenomenon can be attributed to the enhanced mobility of PANI chain segments during thermal imidization, where the treatment temperature exceeded the glass transition temperature of PANI. The increased chain mobility weakened the intermolecular interactions and reduced the packing efficiency of polymer chains, thereby enlarging the free volume within the polymer matrix.

The gas permeation properties of all precursor membranes and derived carbon molecular sieve membranes were evaluated for H_2_, CO_2_, N_2_, and CH_4_ at 35 °C under an upstream pressure of 2 bar. The detailed permeability and selectivity data are summarized in Table 1. As shown in Table 1, all membranes exhibited a consistent permeability sequence of H_2_ > CO_2_ > N_2_ > CH_4_, which agrees well with the kinetic diameters of the corresponding gas molecules. This trend indicates that molecular sieving plays a dominant role in determining gas transport through the membranes [32]. For the PI/PANI precursor membranes, the incorporation of PANI into the pristine PI matrix resulted in simultaneous improvements in gas permeability and selectivity. Moreover, the enhancement in gas separation performance became more pronounced with increasing PANI loading. This behavior can be attributed to the increased FFV induced by PANI incorporation, which provides additional transport pathways for gas molecules and consequently improves permeability. Meanwhile, the interaction between PI and PANI chains may contribute to improved interfacial compatibility and a more uniform microstructural distribution, thereby enhancing the molecular sieving capability and gas selectivity of the membranes. However, when the PANI loading increased to 25%, a significant decline in gas pair selectivity was observed. This deterioration may be attributed to the excessive incorporation of PANI beyond the compatibility limit of the PI matrix. Excessive PANI content can cause PANI aggregation and the formation of non-selective interfacial voids. These structural defects weaken the molecular sieving effect and provide additional non-selective transport pathways, resulting in reduced gas selectivity [33].

After pyrolysis at 650 °C, all CMS membranes exhibited significantly enhanced gas transport properties compared with their corresponding polymer precursors. Notably, the CMS/PANI membranes demonstrated a more pronounced improvement in gas permeability. Compared with the pristine CMS membrane, the CMS/PANI-25 membrane exhibited a remarkable increase in H_2_ permeability from 539 to 2113 Barrer and CO_2_ permeability from 235 to 801 Barrer, corresponding to enhancements of 292% and 240%, respectively. Furthermore, the permeability of all tested gases continuously increased with increasing PANI loading. This improvement can be attributed to the structural evolution induced by PANI incorporation during pyrolysis. The decomposition of PANI promoted the formation of additional microporous structures within the carbon matrix, providing more accessible diffusion pathways for gas molecules. This observation is consistent with the variations in BET surface area and pore size distribution discussed above, further confirming the role of PANI in regulating the porous structure of the carbon framework. In contrast, the H_2_/CH_4_ and CO_2_/CH_4_ selectivities exhibited a volcano-shaped dependence on PANI loading, initially increasing and subsequently decreasing. The maximum selectivities were achieved for the CMS/PANI-20 membrane, with H_2_/CH_4_ and CO_2_/CH_4_ selectivities reaching 253 and 95, respectively. This enhanced molecular sieving performance may be attributed to the optimized ultramicroporous structure generated during pyrolysis, where the narrowed pore apertures effectively restricted the transport of larger CH_4_ molecules while allowing the preferential diffusion of smaller H_2_ and CO_2_ molecules. In addition to the pore size distribution, the local packing and short-range organization of carbon structural domains may also influence the effective dimensions and connectivity of gas transport pathways, thereby contributing to the molecular sieving behavior of CMS/PANI membranes [10]. When the PANI loading was further increased to 25%, the gas selectivity decreased, following a similar trend observed for the PI/PANI precursor membranes. This reduction may be associated with excessive PANI incorporation, which induces the formation of enlarged diffusion pathways and non-selective defects, thereby weakening the molecular sieving capability [34]. These results demonstrate the strong correlation between the precursor structure and the gas separation performance of the derived CMS membranes.

**Table 1:** The gas permeability and selectivity of the separation membranes prepared in this study and other reported CMSMs.

Membrane	Permeability (Barrer)				Ideal Selectivity (α_A/B_)		
	H_2_	N_2_	CH_4_	CO_2_	H_2_/N_2_	H_2_/CH_4_	CO_2_/CH_4_
Pure PI	5.3	0.08	0.05	1.65	66	106	33
PI/PANI-5	6.3	0.07	0.05	1.73	90	126	35
PI/PANI-10	6.8	0.07	0.05	1.94	97	136	39
PI/PANI-15	8.2	0.08	0.06	2.49	103	137	42
PI/PANI-20	11.2	0.11	0.08	3.33	102	140	42
PI/PANI-25	12.7	0.26	0.14	3.9	49	91	28
Pure CMS	539	6.1	2.2	235	88	245	107
CMS/PANI-5	1404	17.24	6.28	488	81	224	78
Aged 30 days	1179	13.96	4.77	400	84	247	84
Aged 60 days	1025	11.07	3.78	351	92	271	93
CMS/PANI-10	1674	19.63	7.01	599	85	239	85
Aged 30 days	1428	15.86	5.32	493	90	268	93
Aged 60 days	1239	12.73	4.03	430	97	307	107
CMS/PANI-15	1861	20.76	7.51	681	89	248	91
Aged 30 days	1567	16.78	5.68	557	93	276	98
Aged 60 days	1385	13.51	4.25	488	103	326	115
CMS/PANI-20	2012	22.6	7.94	754	89	253	95
Aged 30 days	1701	17.46	5.95	616	97	286	104
Aged 60 days	1481	14.02	4.61	540	106	321	117
CMS/PANI-25	2113	27.52	11.87	801	77	178	67
Aged 30 days	1744	21.14	8.57	652	82	204	76
Aged 60 days	1559	17.15	6.53	572	91	239	88
PEESK/PVP-650 [35]	602	29.7	-	357	20.3	-	-
PBI/Matrimid-600 [36]	1337	15.71	5.84	306	85.1	229	52.3
PEI/PEG-650 [37]	-	5.5	3.26	211	-	-	64.7
PI/CA-600 [16]	5300	126	126	-	42	42	-
PBI/PI-600 [38]	-	-	7.27	359	-	-	49.4
PI/550 [39]	675	-	1.69	-	-	399	-

**Figure 7:**
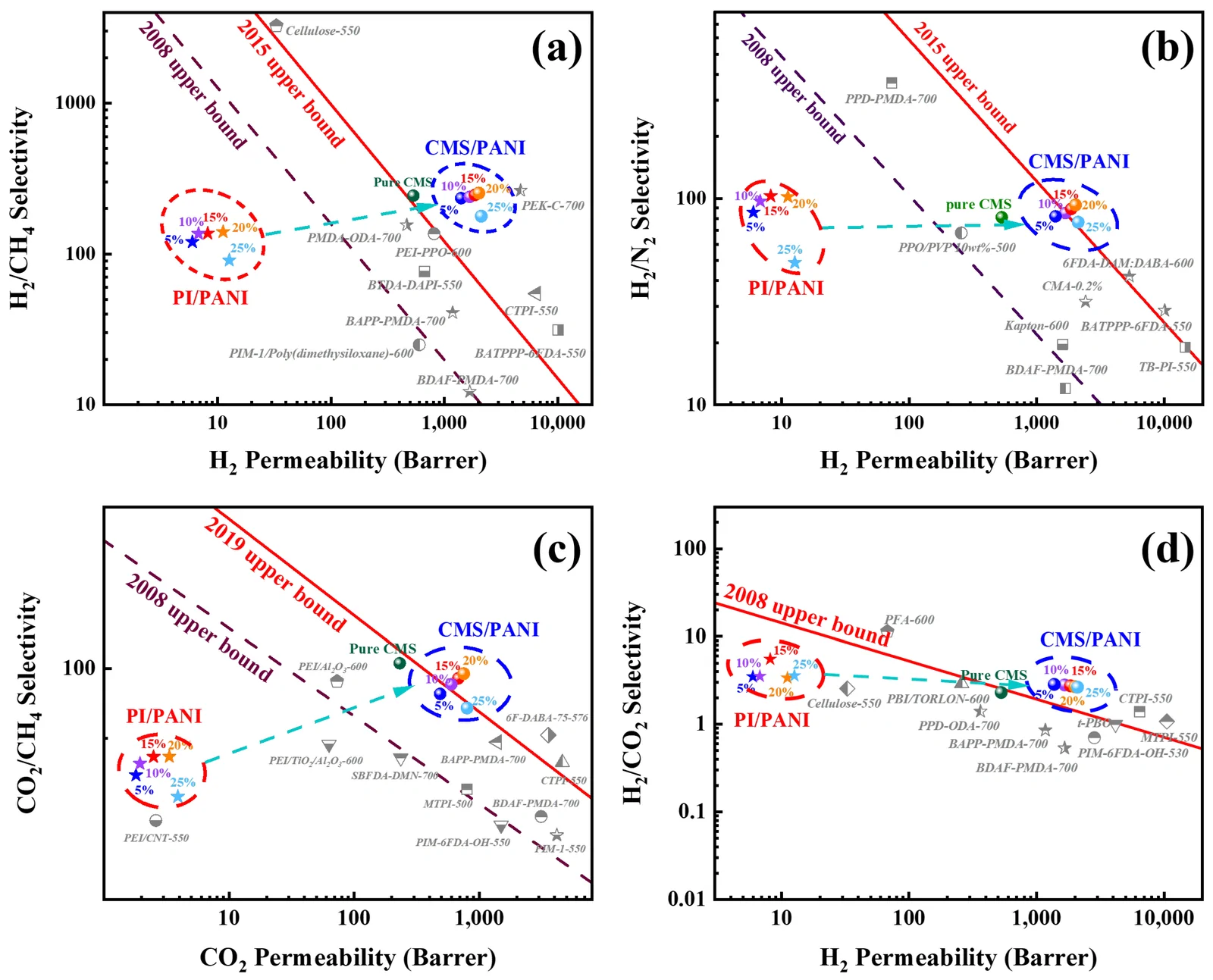
Comparison of the permeability–selectivity performance of CMS/PANI membranes with previously reported membranes and Robeson upper bounds (**a**) H_2_/CH_4_, (**b**) H_2_/N_2_, (**c**) CO_2_/CH_4_ and (**d**) H_2_/CO_2_.

To highlight the competitive separation performance of the CMS/PANI membranes, their permeability–selectivity relationships were compared with those of previously reported state-of-the-art membranes. As shown in Figure 7, the performance of various membranes for H_2_/CH_4_, H_2_/N_2_, CO_2_/CH_4_, and H_2_/CO_2_ separations was evaluated using the corresponding Robeson upper-bound plots.

The results demonstrate that the incorporation of PANI significantly enhanced the gas separation performance of CMS/PANI membranes compared with the pristine CMS membrane. For H_2_/CH_4_ separation, all CMS/PANI membranes surpassed the latest Robeson upper bound, which can be attributed to the formation of abundant microporous and ultramicroporous structures induced by PANI incorporation during pyrolysis. Notably, the CMS/PANI-20 membrane exhibited optimal overall separation performance. It showed excellent separation capabilities for H_2_/N_2_ and H_2_/CO_2_ systems, while its CO_2_/CH_4_ separation performance exceeded the 2019 Robeson upper bound and surpassed that of most previously reported CMS membranes.

Physical aging is an important parameter for evaluating the long-term stability of gas separation membranes and remains one of the critical challenges limiting the practical application of carbon molecular sieve membranes. Generally, physical aging leads to a gradual decrease in gas permeability over time, while gas selectivity remains unchanged or even increases. This phenomenon originates from the thermodynamically non-equilibrium nature of CMS membranes, which tend to undergo structural relaxation toward a more stable state. During this process, the relaxation of distorted carbon chain configurations and rearrangement of the carbon framework can induce the shrinkage or collapse of micropores, ultramicropores, and free volume elements within the membrane structure [40,41,42].

The pure-gas separation performances of CMS/PANI membranes with different PANI contents after aging for 30 and 60 days are summarized in Table 1. Among all membranes, CMS/PANI-25 exhibited the most pronounced physical aging behavior. After 30 days of aging, the H_2_ and CO_2_ permeabilities decreased by 17% and 19%, respectively. After extending the aging time to 60 days, the reductions increased to 26% and 29%, respectively. This behavior can be attributed to the relatively higher BET surface area and more open porous structure of CMS/PANI-25, which provides a greater driving force for pore shrinkage and structural relaxation during aging. Despite the permeability loss, all CMS membranes maintained relatively high gas permeabilities after 60 days of aging, with permeability reductions below 30% for all tested gases. Meanwhile, the gas selectivities increased by approximately 15~35%, remaining at relatively high levels. These results demonstrate that CMS/PANI membranes possess excellent resistance to physical aging, indicating their promising potential for long-term gas separation applications.

**Figure 8:**
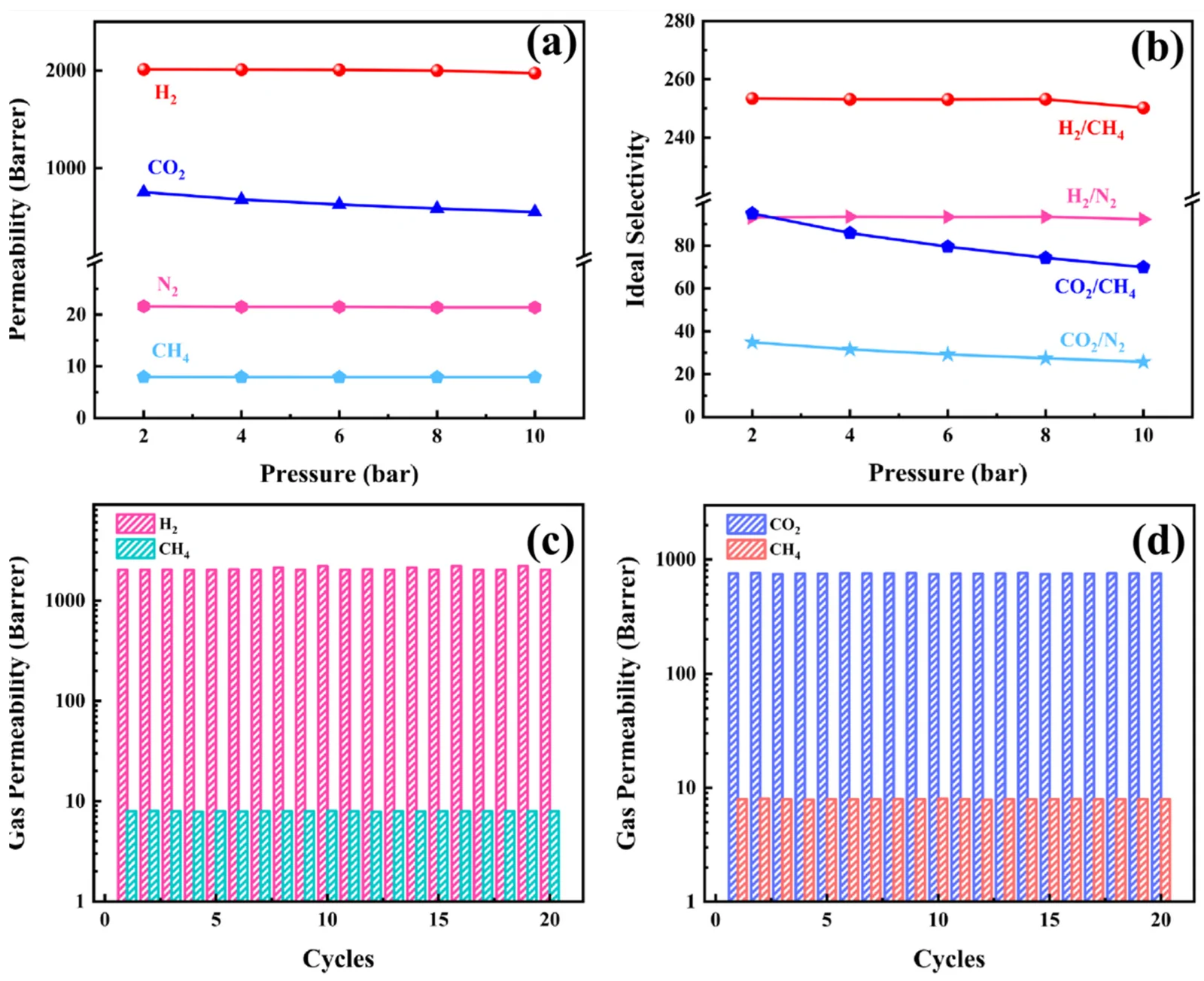
Gas permeability (**a**) and selectivity (**b**) of CMS/PANI-20 under different pressures and cyclic stability tests of CMS/PANI-20 for H_2_/CH_4_ (**c**) and CO_2_/CH_4_ (**d**) gas pairs.

Plasticization resistance is also an important indicator for evaluating the long-term stability of gas separation membranes. Generally, with increasing feed pressure, the permeability of polymer membranes initially decreases and then increases due to the enhanced plasticization effect induced by gas sorption. The pressure at which permeability changes from decreasing to increasing is defined as the plasticization pressure [43]. Generally, a higher plasticization pressure indicates stronger resistance to plasticization. Figure 8a,b shows the gas permeation performance of CMS/PANI-20 under different feed pressures ranging from 2 to 10 bar. With increasing feed pressure, the permeabilities of H_2_, CO_2_, and CH_4_ through CMS/PANI-20 exhibited no obvious increase, and no typical plasticization behavior was observed. This result demonstrates the excellent plasticization resistance of CMS/PANI-20, which can be attributed to the rigid carbon framework formed during pyrolysis. The rigid structure effectively suppresses gas sorption-induced structural relaxation and pore expansion, thereby maintaining stable molecular sieving properties. Furthermore, during 20 consecutive cyclic tests, the gas permeability of CMS/PANI-20 showed only slight fluctuations, indicating its excellent pressure stability and good reproducibility for repeated gas separation operations.

## 4. Conclusions

In this study, a PI/PANI blending strategy was developed to regulate the microstructure and gas separation performance of polyimide-derived carbon molecular sieve membranes. The incorporation of PANI effectively increased the fractional free volume of the PI/PANI precursor membranes and promoted the development of microporous and ultramicroporous structures during pyrolysis, resulting in a more accessible porous network for gas transport. With increasing PANI content, gas permeability was significantly enhanced, while an optimal balance between permeability and selectivity was achieved at a PANI loading of 20 wt%. The CMS/PANI-20 membrane exhibited H_2_ and CO_2_ permeabilities of 2012 and 754 Barrer, respectively, together with H_2_/CH_4_ and CO_2_/CH_4_ selectivities of 253 and 95. The present study reveals a close relationship between PANI-induced changes in precursor free volume and the subsequent development of microporous structures during pyrolysis, providing new insight into the structural regulation of PI-derived CMS membranes. In addition, the optimized CMS/PANI membrane exhibited good resistance to physical aging and plasticization, demonstrating its potential for long-term gas separation applications. Overall, PANI-assisted precursor engineering provides an effective approach for developing high-performance carbon molecular sieve membranes with improved permeability, selectivity, and stability.

## Data Availability

The raw data supporting the conclusions of this article will be made available by the authors upon request.
